# Event and Comment

**Published:** 1911-11-15

**Authors:** 


### DENTAL REGISTER.

Vol. LXV. November 15, 1911. No. 11

### EVENT AND COMMENT.

THE CAST INLAY PATENT SITUATION. We are printing in this issue two papers which probably put the Cast Inlay Patent Situation before the profession more clearly and up-to-date than anything heretofore published. There has been during the past six months considerable discussion as to the action of the officers of the Dental Protective Association in giving their approval to Taggart's patent claims, because of the improper influence of such an action in prejudicing the suit of Taggart against a Washington dentist for infringing his patents. We do not wish to aid or influence this case in the courts by an unwarranted publicity, and do not believe we are doing so by publishing these papers; because we realize that this matter will be adjusted in time by the profession regardless of the court's decision. We print these articles because they seem to represent the sentiment as well as the actual position taken by the men most directly interested in the controversy, and who ought to be able to present the Taggart side of the controversy in its strongest terms. The profession will then be able to form its own opinions as to where it ought to stand on the question and individuals will be able to intelligently credit or discount as they may see fit the positions taken by Taggarts strongest supporters. We trust our readers may be sufficiently interested to read Dr. Crouse's letter, that they may form their own opinions on an intelligent basis as where their duty lies. That this question may be disposed of as soon as possible is the common desire of all right thinking members of our profession.

IMPROVED PLATE PROSTHESIS. In view of the fact that large numbers of the best dental practitioners have either quit making artificial dentures, or make them only by proxy,—as through the commercial laboratories,— we are sometimes inclined to believe that plate prosthesis will eventually become one of the lost arts, so far as the better class of practitioners are concerned. But those who read carefully and give any time to the study of what is being done for Prosthodontia in several parts of the world, know that the subject of right articulation and scientific occlusion is being most thoroughly studied by some of the most earnest men in our profession and that real advancement in knowledge and skill can be recorded. A most significant fact, that the profession has not lost its interest in this branch of practice may be recorded in the announcement that hereafter all candidates wishing to come up for examination and registration for practice in the State of Illinois, must be prepared to stand an examination on modern dental plate prosthesis, both theoretical and practical. They will be required to present upper and lower dentures mounted on a modern anatomical articulating frame demonstrating the latest ideas of articulation and occlusion. If this test is faithfully carried out we predict that some changes will need to be made by some schools in their methods of instruction on this subject. The researches of Gysi, Williams and others on the subject of articulation and anatomic tooth forms also leads us to believe that plate prosthesis is still one of the essentially live topics in technical dentistry.

